# Egocentric and Allocentric Spatial Memory for Body Parts: A Virtual Reality Study

**DOI:** 10.5334/joc.357

**Published:** 2024-04-15

**Authors:** Silvia Serino, Daniele Di Lernia, Giulia Magni, Paolo Manenti, Stefano De Gasperi, Giuseppe Riva, Claudia Repetto

**Affiliations:** 1 Department of Psychology, Universitàdegli Studi Milano –Bicocca, Milan, Italy; 2 Humane Technology Lab, UniversitàCattolica del Sacro Cuore, Milan, Italy; 3 Faculty of Psychology, UniversitàCattolica del Sacro Cuore, Milan, Italy; 4 Department of Computer Science, University of Pisa, Pisa, Italy; 5 Applied Technology for Neuro Psychology Lab, IRCCS Istituto Auxologico Italiano, Milan, Italy; 6 Department of Psychology, UniversitàCattolica del Sacro Cuore, Milan, Italy

**Keywords:** spatial memory, egocentric memory, allocentric memory, body, virtual reality

## Abstract

Extensive literature elucidated the mechanisms underlying the ability to memorize the positions of objects in space. However, less is known about the impact that objects’ features have on spatial memory. The present study aims to investigate differences in egocentric and allocentric object-location memory between hand stimuli depicted in a first-person perspective (1PP) or in a third-person one (3PP). Fifty-two adults encoded spatial positions within a virtual museum environment featuring four square buildings. Each of these buildings featured eight paintings positioned along the walls, with two pictures displayed on each of the four walls. Thirty-two stimuli were employed, which represented pictures of the right hand performing various types of gestures. Half of the stimuli depicted the hand in the 1PP, while the other half depicted the hand in the 3PP. Both free and guided explorations served as encoding conditions. Immediately after that, participants underwent a two-step object-location memory task. Participants were provided with a map of the museum and asked to identify the correct building where the image was located (allocentric memory). Then, they were presented with a schematic representation of the exhibition room divided into four sections and instructed to select the section where they thought the picture was located (egocentric memory). Our findings indicate a memory performance boost associated with egocentric recall, regardless of the perspective of the bodily stimuli. The results are discussed considering the emerging literature on the mnemonic properties of body-related stimuli for spatial memory.

## 1. Introduction

Remembering where things are located – memory for object locations – is of vital importance for most of our daily activities. We are immersed in a world of objects, and remembering their spatial locations is critical for planning actions, orienting ourselves, and effectively navigating our surroundings (Ekstrom & Hill, 2023; Manns & Eichenbaum, 2009; Postma & De Haan, 1996). Several experimental studies have been conducted to elucidate the mechanisms underlying the ability to memorize the positions of objects (Postma, Kessels, & van Asselen, 2008) and the magnitude of individual differences in object-location memory tasks (Voyer, Postma, Brake, & Imperato-McGinley, 2007). However, less is known about the impact that objects’ features have on spatial memory.

The literature initially explored the extent of gender differences and proposed that the characteristics of the object to be remembered (common, uncommon, gender-neutral, geometric, masculine, feminine) could indeed impact the memory of spatial locations (see for example, (McGivern et al., 1997; Voyer et al., 2007). Moreover, emotion and memory are also known to interact, with arousing stimuli being recalled better than neutral ones (Hamann, 2001). The enhanced memory for emotional items occurs also when people are asked to memorize their spatial locations, as found by some authors (Mather & Nesmith, 2008). In their study, participants were asked to memorize (through an incidental encoding task) the spatial locations of positive and negative arousing pictures. Results indicated that participants recalled the positions of arousing items better than non-arousing ones, independently of their valence. In a series of three online experiments, Babo-Rabelo and collaborators (2022) have recently explored the mechanisms that connect the aesthetic experience of a painting with the recollection of its spatial location. In their study, participants first navigated in a virtual museum to incidentally encode the spatial locations of 24 paintings. Then, they were asked to perform an object-location memory task requiring them to identify the exhibition room in which the painting was seen (i.e., first to fourth) and the correct wall (left, front, or right wall relative to the entrance door). Importantly, recalling the exhibition where the painting was displayed might suggest the capacity to construct an allocentric, world-centered map of the environment. Conversely, remembering the specific wall might imply the ability to create an accurate body-based egocentric representation (Marchette, Vass, Ryan, & Epstein, 2014). Finally, participants were asked to rate how much they liked each painting. Results indicated that positive aesthetic experiences of paintings were associated with an enhanced memory of the wall where the object was located, suggesting a link between affect and body-based egocentric memory (Babo-Rebelo et al., 2022).

Furthermore, neuroimaging and behavioral studies suggest that *bodies* are the most special of the objects we are surrounded by (Repetto & Riva, 2023; Riva, 2018; Slaughter et al., 2004). This peculiarity is relevant also in the memory processes, with behavioral and neuroimaging studies on working memory demonstrating that body-related stimuli have unique mnemonic features (Galvez-Pol, Calvo-Merino, & Forster, 2020; Galvez-Pol, Forster, & Calvo-Merino, 2020). Evidence collected so far suggests that bodily stimuli are encoded and stored by recruiting, at least partially, specific memory systems and resources. Numerous studies, spanning behavioral and neuroimaging investigations into action observation (Galvez-Pol, Calvo-Merino, Capilla, & Forster, 2018; Wood, 2009) to those focused on action recognition (Smyth, Pearson, & Pendleton, 1988; Smyth & Pendleton, 1990), concur that our sensorimotor system plays a critical role in the encoding, retention, and recall of body-related stimuli. Studies have also shown that memory for bodily stimuli is usually impaired by increasing the body-related simultaneous load with a secondary sensorimotor task (Cortese & Rossi-Arnaud, 2010; Vicary & Stevens, 2014). In addition, recent studies revealed a modulation in somatosensory activity during encoding and maintenance of hand images in a working memory task (Galvez-Pol et al., 2018), along with observed motor activity modulation during the encoding of these hand images (Galvez-Pol, Forster, & Calvo-Merino, 2018), highlighting the presence of both somatosensory and motor modulations.

Building on this literature, the current study aims at exploring the role of bodily stimuli in spatial memory. To this aim, we developed a novel immersive virtual reality scenario representing a museum area with four different squared buildings. During the experiment, the participants navigated in the virtual museum to intentionally encode the spatial locations of 32 paintings with a free and guided exploration (Marchette et al., 2014). Each building contained two paintings hung up on each of the walls (i.e., eight paintings in total for each building). All paintings represented pictures of the right hand performing various types of gestures shown in two different perspectives: half of the stimuli depicted the hand in the first-person perspective (1PP), and half of the stimuli depicted the hand in the third-person perspective (3PP). The first objective of the study was to investigate potential differences in the recall of spatial locations for bodily stimuli when presented in a 1PP vs. 3PP. A consistent body of evidence demonstrated indeed that different neural responses and cognitive processing are active during the processing of hand stimuli presented in 1PP vs. 3PP (Chan, Peelen, & Downing, 2004; Maeda, Kleiner-Fisman, & Pascual-Leone, 2002; Saxe, Jamal, & Powell, 2006). Hand stimuli presented in 1PP are motorically familiar and typically processed by relying on sensorimotor simulation of one’s body movements; hands presented in 3PP are instead difficult to assume and typically processed by switching from sensorimotor to visual processing (Vogt, Taylor, & Hopkins, 2003). Studies have found differences in brain activity in response to bodily actions or images of identical body parts, depending on whether these were presented in the 1PP or 3PP (Ge et al., 2018; Saxe et al., 2006). Specifically, bodily stimuli presented in 1PP elicit greater activation not only in extrastriate areas but also in sensorimotor areas (Ge et al., 2018; Saxe et al., 2006).

The second objective of the study was to explore differences in the accuracy of egocentric and allocentric recall for spatial locations of bodily stimuli. Spatial memory performance was evaluated with an object-location memory task adapted from Babo-Rebelo et. al (Babo-Rebelo et al., 2022). Participants were asked to identify the correct building (i.e., allocentric memory) and the side of the building on which the painting was hung (i.e., egocentric memory). Robust evidence in the literature suggested indeed that hippocampal regions support allocentric world-centered representations, while parietal areas contribute to egocentric body-centered ones (Bottini & Doeller, 2020; Burgess, 2002; Byrne, Becker, & Burgess, 2007). In particular, egocentric representations rely on the movement of the observer in the environment and are closely linked to action preparation and sensorimotor information (Burgess, Maguire, & O’Keefe, 2002).

In relation to the two objectives, we made the following hypotheses. First, considering the reviewed role of the sensorimotor system for body-related stimuli (Galvez-Pol, Forster, et al., 2020), and based on previous literature that bodily stimuli presented in 1PP elicit greater activation in sensorimotor areas (Chan et al., 2004; Ge et al., 2018; Maeda et al., 2002; Saxe et al., 2006), we predicted that bodily stimuli presented in 1PP will result in improved memory performance compared to those presented in 3PP (H1). In addition, we predicted that bodily stimuli presented in a 1PP, as opposed to a 3PP, would exert a preferential impact on egocentric memory as opposed to allocentric one (H1a). Second, considering the role of the sensorimotor system in the construction of egocentric representations (Byrne et al., 2007), we hypothesized that bodily stimuli would have a preferential effect on egocentric memory compared to the allocentric one (H2).

## 2. Methods

### 2.1 Participants

Fifty-two participants (59.6% females, mean age = 23.5, SD = 2.7, range: [20, 37]) took part in the study. We conducted a power analysis using G*Power (Faul, Erdfelder, Lang, & Buchner, 2007) to determine the required sample size for our repeated measures design. Due to difficulties in conducting a power analysis on a multilevel model, we used a small effect size with a similar design and with similar levels, focusing on within-subjects’ factors. The analysis was performed a priori, based on Cohen’s (1988). The power analysis was calculated with an anticipated effect size f of 0.20, which is considered a small effect size according to Cohen (1988). The alpha error probability was set at 0.05, and the desired power (1-β error probability) was targeted at 0.8. We structured our design around 2 groups with 2 measurements each, assuming a correlation among repeated measures of 0.5 and a nonsphericity correction ɛ of 1, which is suitable for designs with equal variances across conditions. The output from G*Power indicated a noncentrality parameter λ of 8.32, with a critical F-value of 4.0343097. The analysis suggested that a total sample size of 52 would be required to achieve an actual power of approximately 0.8074866.

Participants were recruited through advertisements at Università Cattolica del Sacro Cuore, where the experiment took place, and through personal contact. All the participants had normal or corrected-to-normal vision and no history of neurological and psychiatric diseases. The participants were naïve to the aims and hypotheses of the study. The study was approved by the University’s Ethics Committee (protocol number 42–22). All participants gave written consent before testing and received a voucher worth 10 euros as compensation for their participation.

### 2.2 Apparatus and stimuli

#### 2.2.1 The virtual environment

The virtual environment was designed in Unity (Unity Technologies, CA, USA). It represented a park containing four squared museums, as in Marchette and co-authors (Marchette et al., 2014). Each building had the same structure, but it was painted with different colors (blue, red, yellow, and green) and was located at the end of a cross-shaped path; the starting point was located at the crossroad, facing the blue museum. Orientational cues surrounded the park, such as apartment buildings and mountain range.

Each museum contained eight paintings arranged along the walls, with 2 pictures hung up on each of the 4 walls. Footprints were drawn in front of each picture to invite the participants to position themselves to observe the painting only from a specific direction (i.e. facing the painting).

#### 2.2.2 Hand stimuli

Sixteen gestures representing different hand positions were selected, performed only with the right hand. We strived to exclude explicit iconic gestures and symbolic gestures to avoid potential connections to conceptual representations (both concrete and abstract meanings) that could affect their memorization (D’Argembeau & Van der Linden, 2004; Macedonia, Müller, & Friederici, 2011). However, given the large number of different gestures to depict, we cannot rule out that some of the gestures may have had symbolic meaning.

**Figure 1:**
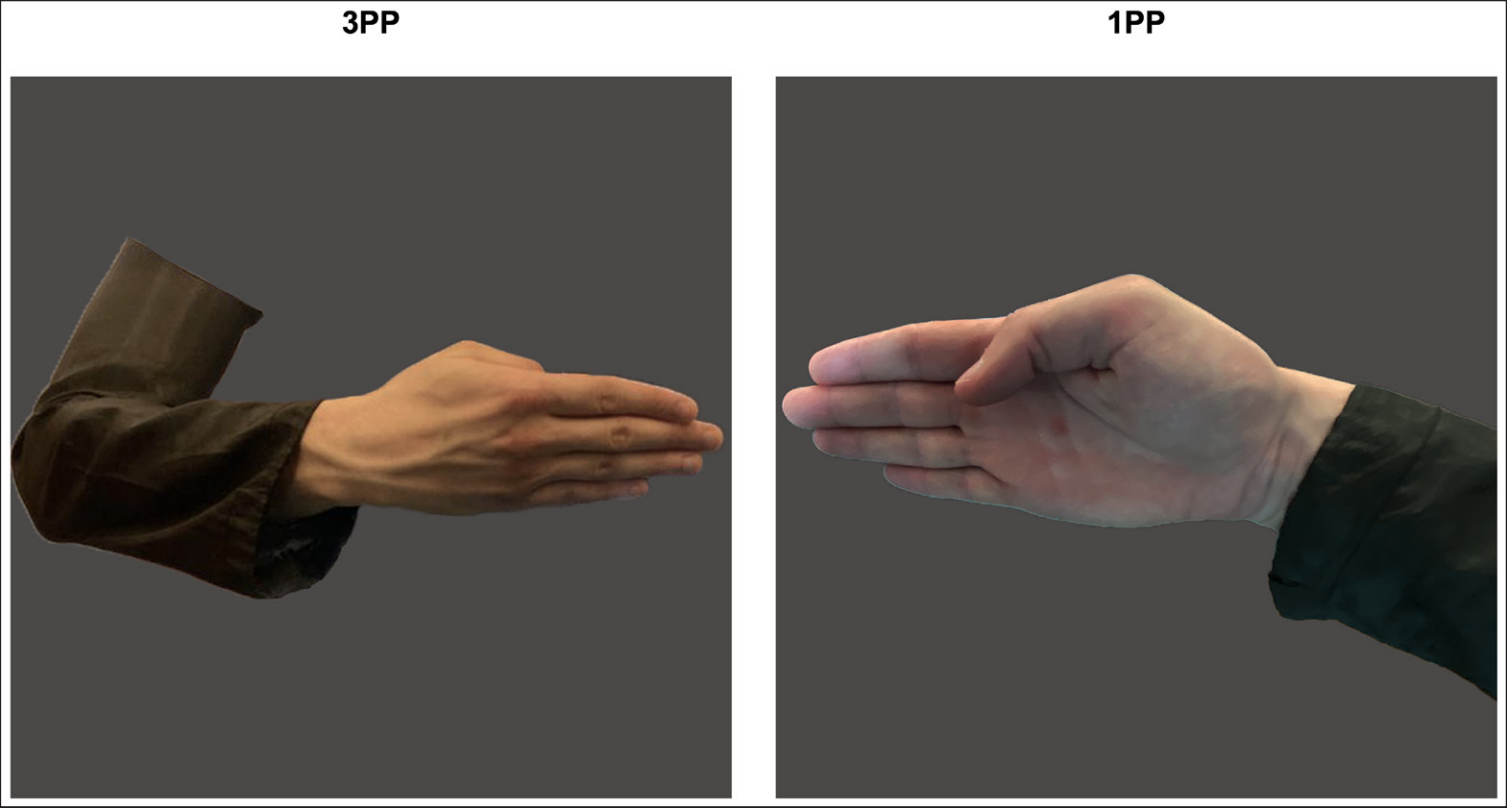
**Hand pictures**. An example of the stimuli used: the same gesture is depicted from 3PP (left panel) and from 1PP (right panel).

For each gesture, 2 pictures were taken from 2 different perspectives: first-person perspective (the depicted hand could belong to the observer as it was displayed in a position compatible with the observer’s point of view – 1PP), and third-person perspective (the depicted hand could belong to an individual located in front of the observer – 3PP). The pictures were taken simultaneously from both perspectives to represent the very same position. The individual performing the gestures was a young man wearing a black shirt. All the accessories were removed. The pictures included only the hand and the forearm. Overall, the set of stimuli comprised 32 pictures, 16 depicted from 1PP of view and 16 depicted from 3PP (see an example in Figure 1). All the pictures were displayed in color against a grey background, at a resolution of 1024 × 1024 pixels. The images were assigned to the four museums, making sure that each museum included 4 1PP-photos and 4 3PP-photos. Within each exhibition room, the location of the 8 pictures was randomized across participants.

### 2.3 Procedure

#### 2.3.1 Encoding phase

The encoding phase took place within the virtual environment and followed the paradigm used by Marchette and collaborators (2014). The participants were welcomed in a quiet room by an experienced researcher, and after having read and signed the informed consent, wore the head-mounted display (Oculus Rift) connected to the laptop running the virtual environment. After having received instructions on how to navigate within the environment using the controllers, the virtual experience started, with a brief familiarization phase allowing the participants to put the instructions into practice. In this phase, the participants entered an environment like that of the encoding phase but simplified: the scenario was colored only in gray scale and the starting point was a small square where a short walkway began, which led to a single building. The experimenter prompted the participant to move using the controllers, to enter the building where only one picture was hung on the front wall. The experimenter illustrated to the participant how to validate the picture once it has been found (i.e. standing in the correct location facing the picture and pressing a key on the controllers). Specifically, the participant was instructed to position themselves in front of the stimuli at a designated spot marked with two footprints on the floor. Once the participant felt confident, the encoding phase started. This phase was divided into a free exploration (i.e., without a specific target to localize) and a guided learning period (i.e., with a specific target to localize). This approach seemed particularly suitable for our purposes as it allowed a deep encoding of the pictures, thanks to the deployment of different strategies: during the free exploration, the participant could use individual strategies to explore and remember the object locations, while during the guided exploration the participant’s attention was drawn to each of the picture location one at the time to strengthen its memorization.

During the free exploration, participants, starting from the crossroad, were told to enter one museum at a time (in randomized order), and to explore the exhibition room and the included pictures, trying to remember their correct locations. For each building, 1 minute of free exploration was allowed, after which the participant was invited to exit the museum and enter the next one. This phase lasted 4 minutes.

During the guided learning period, starting again from the initial position (the middle of the crossroad), with one picture appearing at a time. The task was to find the picture within the museums and try to remember its spatial location. The participants were free to explore the museums at their will until they found the matched picture. During the search, they could press the right controller to have the to-be-found picture displayed again, with no limitations. Once the correct location was identified, the participant had to point with the right controller to the picture, to validate the localization. Once the answer was registered, they were teleported back to the starting position, and the next stimulus appeared, and the participant started the search again, up to the 32nd stimulus, after which the virtual task ended. As in Marchette and collaborators (Marchette et al., 2014), all pictures remained visible during this phase to provide additional opportunities for participants to encode their positions. In both encoding tasks, participants were explicitly told to try to remember the spatial locations of the bodily stimuli. No time limit was set up for the guided exploration phase. On average, participants spent 52.66 seconds (SD=40.58) for each encoding trial [1PP: mean 51.94 ± 43.35, 3PP: 53.38 ± 37.58], resulting in an average duration for the guided exploration phase of 28.08 minutes.

#### 2.3.2 Object-location memory tasks

**Figure 2:**
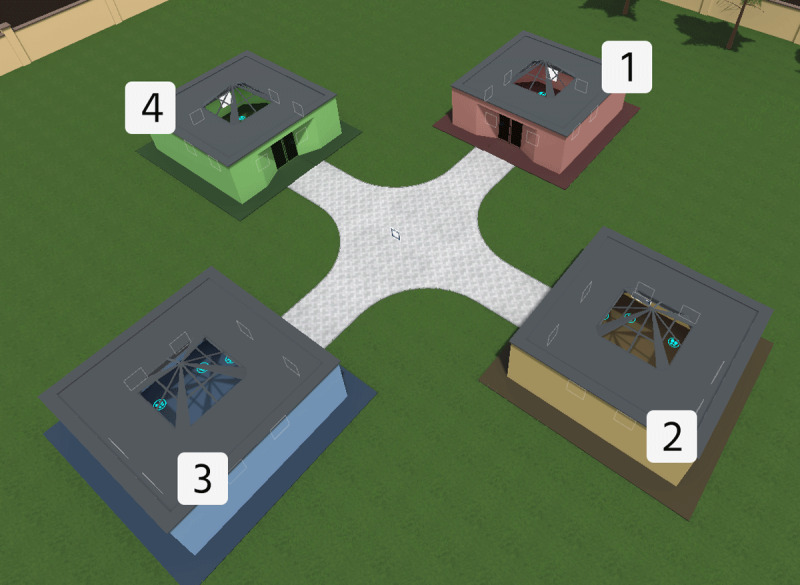
**The map of the museum area**. This map was provided to the participants during the allocentric memory task and shows the four colored buildings and a number associated with each of them.

**Figure 3:**
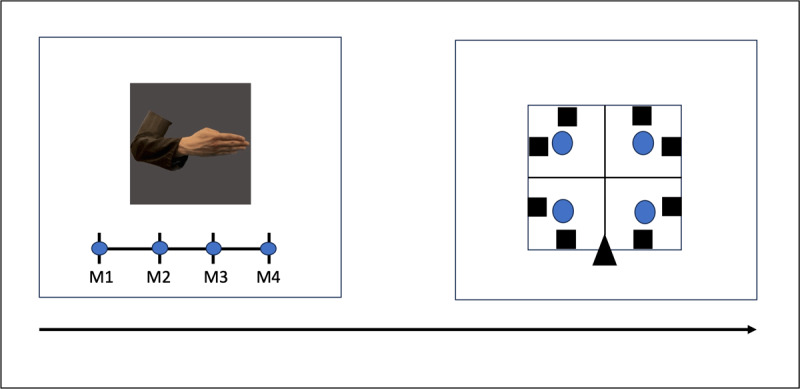
**The experiment procedure**. In the allocentric memory task (left panel) the participant was asked to select one of the four options in the bottom line (M1 to M4, indicating the four museums); in the egocentric memory task (right panel) the participants had to select one of the four blue dots to indicate in which room section the picture was located.

Immediately after the encoding phase, participants underwent a two-step memory task, run on Psychopy (v2022.2.5) (Peirce et al., 2019). Participants were presented with a picture in the middle of the screen; below, there was a line with 4 bars indicating the museums from 1 to 4 (M1- M2- M3- M4). The participants were instructed to click on the bar indicating the museum where they thought the picture was located. For this task participants were provided with a map of the museums with the colored buildings and a number associated with each of them (Figure 2 – allocentric memory task). After the response was registered, in the middle of the screen a schematic representation of the exhibition room appeared. A black triangle represented the door position, and, on each wall, black squares indicated the pictures’ position. The room was ideally divided into 4 sections, including 2 pictures each. In addition, blue circles indicated the point to click to select the correspondent room section. Participants were instructed to select the room section where they thought the picture was located (egocentric memory task). In this task, the experimental setup would have allowed us to collect a more precise measure of the egocentric memory, mainly the specific location of the picture within the room (1 out of 8 options) instead of the current measure (1 out of 4 options). However, we chose the current measure for two reasons: first, a pilot study had indicated that the task with 8 options was far too difficult, leading to a poor performance; second, the division of the room in 4 sections allowed to directly compare the two tasks (egocentric vs allocentric) as both offer the same number of options. After the response was given a new stimulus was presented. The pictures were presented in random order. No time limit was set for any of the tasks. In both tasks, we measured participants’ accuracy, namely correctly remembered spatial locations of hand stimuli. Correct responses were coded as 1 and errors were coded as 0. The entire procedure is illustrated in Figure 3.

### 2.4 Data Analysis

A generalised linear mixed-effects model, specifying a binomial distribution and logit link function, was fit to the accuracy data of participants; this was performed in R (RStudio Team, 2015) using the glmer function in lme4 (Bates, Mächler, Bolker, & Walker, 2015).

The model was estimated through maximum likelihood estimation using the Nelder-Mead optimiser. Two dummy-coded predictors were entered into the model: Perspective (0 = first-person, 1 = third-person) and Memory Type (0 = allocentric, 1 = egocentric) along with their interaction. Additionally, one continuous variable representing encoding time during the free exploration task was incorporated as a control variable; this encoding time variable was z-scored to facilitate model convergence. Two random intercepts were included in the model, one for each participant and the other for each stimulus. Estimated marginal means were calculated using the emmeans package (Lenth, 2023) and back-transformed from logit space. Linear functions of predictors were estimated using joint_tests. Model assumptions were evaluated using the performance package (Lüdecke et al., 2021). No outliers or participants were removed from the analysis.

## 3. Results

**Figure 4:**
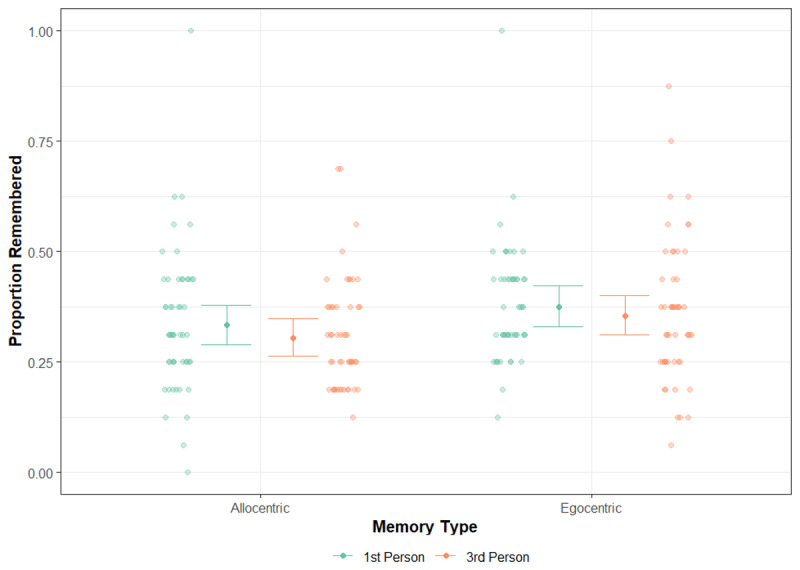
**The proportion of stimuli remembered split by memory type (allocentric and egocentric) and the perspective of the stimulus (first-person and third-person)**. The error bars show the mean and 95% confidence intervals around each estimate. The jittered points beside each error bar show the mean accuracy of participants on the task across each of the conditions.

In Figure 4, the impact of memory type and stimulus perspective on the proportion of items remembered by participants is illustrated. Our statistical analysis revealed a significant main effect of memory type, χ^2^ (1) = 7.83, p = .005, indicating higher accuracy in the egocentric condition (M = 0.37, SE = 0.02) compared to the allocentric condition (M = 0.32, SE = 0.02). However, there was no significant difference in accuracy between stimuli presented in the first-person (M = 0.35, SE = 0.02) or third-person (M = 0.33, SE = 0.02) perspective, χ^2^ (1) = 1.34, p = .247. Additionally, no significant interaction was observed between memory type and perspective, χ^2^ (1) = 0.10, p = .758. The analysis did find that encoding time had a significant effect, with increased exploration being linked to lower memory accuracy, χ^2^ (1) = 15.03, p < .001. However, the inclusion of this covariate did not alter the outcomes of the other effects, with the same conclusions being reached with or without its inclusion in the model. More detailed information about the model summary is available in the Supplementary Information (Table S1).

## 4. Discussion

In this study, we used a VR-based scenario to investigate potential differences in egocentric and allocentric object-location memory between hand stimuli depicted in a first-person perspective (1PP) or in a third-person one (3PP).

Our findings did not indicate any significant difference in the retrieval of stimuli between 1PP and 3PP (H1). Furthermore, there was an absence of an additional memory benefit for 1PP stimuli in the egocentric memory task (H1a). Indeed, on the basis of literature suggesting that hand stimuli presented in this perspective can benefit from sensorimotor simulation of one’s own body movements (Chan et al., 2004; Maeda et al., 2002; Saxe et al., 2006; Vogt et al., 2003), we predicted an enhancement in memory performance after encoding spatial locations in 1PP. The absence of a memory advantage for first-person body stimuli in the present experiment may be due to the encoding task used, which involved searching within a virtual environment. In previous work where a 1PP advantage has been found, participants processed stimuli without the need for active navigation (Ionta & Blanke, 2009; Parsons & Fox, 1998). Although participants were instructed to position themselves in front of the stimuli at a designated spot marked with two footprints on the floor, in future studies, it would be advantageous to explore the potential advantages of a static presentation of stimuli without requiring active encoding.

In line with our hypothesis (H2), our results indicated that memory performance was better for the egocentric retrieval task as compared to the allocentric one. The improved spatial memory performance in egocentric retrieval for bodily stimuli is consistent with literature indicating a close association between these spatial representations and sensorimotor information, as the ability to locate an object relative to one’s body is crucial for subsequent actions (Byrne et al., 2007; Maguire et al., 1998). For instance, Wang and colleagues (Wang, Taylor, & Brunyé, 2012) found that college students with experience in navigating a campus were more likely to activate perceptual-motor association when recalling spatial memories from an egocentric perspective. Therefore, the present findings add to both the debate about which features of an object influence spatial memory and the literature suggesting that bodies have unique mnemonic characteristics.

Finally, it is important to note that most experiments contributing to the current understanding of spatial cognition and object-location memory have been carried out in non-ecological settings. In our daily life, spatial navigation instead takes place in enriched environments, where *bodies* play an essential role (Epstein, Patai, Julian, & Spiers, 2017; Huffman & Ekstrom, 2021). Immersive VR is an excellent instrument for studying human spatial memory due to its capability to immerse users in a digitally realistic and fully controlled environment. The navigation tasks in virtual worlds can involve a diverse set of strategies, engaging multiple sensory modalities and brain regions, thereby creating an embodied spatial experience (Jeung, Hilton, Berg, Gehrke, & Gramann, 2022).

Furthermore, the advent of the Metaverse (Riva and Wiederhold, 2022), will open new possibilities to investigate the mechanisms involved in processing the spatial locations of other individuals within an environment (Diersch & Wolbers, 2019). Literature has predominantly focused on objects as landmarks or spatial targets (Byrne et al., 2007). However, in our social environment, encoding another person’s spatial position is equally crucial for daily life and navigating our surrounding (Kuehn, Chen, Geise, Oltmer, & Wolbers, 2018). In these digital social spaces, we may encounter individuals embodying avatars, experienced from either the 1PP or the 3PP. For future studies, this will provide an excellent testing environment for investigating the processing and object-location memory of the spatial positions of others in our environment.

### 4.1 Constraints of Generality Statement

The stimuli consisted of pictures representing only hands, displaying different gestures, depicted from the 1PP or 3PP. We expect the results to generalize for other hand pictures, provided that the gestures are not too explicitly symbolic or iconic, as in this latter case the gesture meaning could affect its retention, hindering the possibility to specifically understand the role of the memory task and the picture perspective. However, we predict also that the same results would be obtained with other body-related pictures (e.g. legs). The study involved young adults sampled mainly at the Università Cattolica Campus, but we have no reason to believe that any specific characteristic of the participants or the context could have influenced the results. We therefore expect that the same findings could be replicated in different countries and languages, recruiting adult participants.

## Data Accessibility Statement

The data obtained from this study and the stimuli used are available at the following link: 10.17605/OSF.IO/92JX6.

## Additional File

The additional file for this article can be found as follows:

10.5334/joc.357.s1 Table S1. Detailed information about the model summary.
