# Supplementary material for: Egocentric and Allocentric Spatial Memory for Body Parts: A Virtual Reality Study
Source: J Cogn. 2024 Apr 15;7(1):33. doi: 10.5334/joc.357 (PMC11025578; doi:10.5334/joc.357)
Supplement: Table S1. — Detailed information about the model summary. [file joc-7-1-357-s1.pdf]

Supplementary Information

Table S1.
Model summary of generalised linear mixed effects model.

Fixed Effects				
**Term**	**Estimate**	***SE***	***z***	***p***
Intercept	-0.69	0.10	6.83	< .001
Memory Type: Egocentric	0.19	0.10	1.78	.076
Perspective: Third Person	-0.13	0.12	1.09	.275
Encoding Time (Z-Scored)	-0.17	0.04	3.88	< .001
Memory Type x Perspective	0.05	0.15	0.31	.758
**Random Effects**				
**Term**	**Variance**	**SD**		
Participant (Intercept)	0.14	0.38		
Stimulus (Intercept)	0.03	0.17		
**Model Fit**				
**Marginal R^2**	0.01			
**Conditional R^2**	0.06			
**AIC**	4232.44			
**BIC**	4275.21			

Notes. Model reported in logit space.
Model equation: Accuracy ~ Memory * Perspective + Encoding Time + (1 | Participant) + (1 | Stimulus)
